# Utility of cardiac magnetic resonance imaging in the diagnosis of cardiac sarcoidosis

**DOI:** 10.1186/1532-429X-16-S1-P258

**Published:** 2014-01-16

**Authors:** Madhusudan Ganigara, Kelly Stanton, Tamera Corte, Peter Corte, Paul Torzillo, Raj Puranik

**Affiliations:** 1 The Children's Hospital at Westmead, The University of Sydney, Sydney, New South Wales, Australia; 2 The Royal Prince Alfred Hospital, The University of Sydney, Sydney, New South Wales, Australia

## Background

Cardiac involvement in systemic sarcoidosis occurs in 20-25% of patients; and is associated with poor outcome. Identifying cardiac sarcoidosis (CS) is often clinically challenging. Gadolinium-enhanced cardiac magnetic resonance imaging (CMR) is emerging as an important modality in the diagnosis of CS. We sought to determine the utility of CMR in the diagnosis of CS.

## Methods

We retrospectively evaluated patients with known pulmonary sarcoidosis who were referred for CMR at 1.5T as part of evaluation for CS. We reviewed electrocardiograms, 24-hour Holter studies, echocardiograms, and where available gallium and PET scans in these patients. The diagnostic accuracy of CMR for CS was compared to the 1993 Japanese Ministry of Health and Welfare guidelines (JMHW).

## Results

Nine (24%) of 37 patients (49 +/- 14 years; 53% Male) had CMR findings consistent with CS. All of them had late gadolinium enhancement (LGE) predominantly involving the basal septal and lateral segments of the left ventricular myocardium. This distribution of fibrosis did not reflect a vascular cause. Seven of the nine CMR positive patients had changes suggestive of edema/inflammation in the affected myocardial segments on T2 weighed imaging. Only 2 of the 9 patients with CMR positivity met the JMHW criteria for CS. Of the 28 patients who were negative for CS on CMR, one met JMHW criteria for CS. There was some association noted between the stage of pulmonary sarcoidosis and CS positivity on CMR - CS was rare in those with isolated lymph node involvement (11%) and was more common in patients with stage III or IV disease (67%). However, LGE on CMR was seen across all four pulmonary stages.

## Conclusions

CMR has a higher sensitivity and may have more optimal specificity for the diagnosis of CS compared to the JMHW diagnostic criteria. We suggest a greater role for CMR in the diagnosis of CS.

## Funding

None.

